# Supplementary material for: Characterization of Sodium Channel Peptides Obtained from the Venom of the Scorpion Centruroides bonito
Source: Toxins (Basel). 2024 Mar 1;16(3):125. doi: 10.3390/toxins16030125 (PMC10974992; doi:10.3390/toxins16030125)
Supplement: Supplementary file 1 [file toxins-16-00125-s001.zip › toxins-2759276-supplementary.pdf]

toxins

MDPI

Supplementary Materials: Characterization of Sodium Channel Peptides Obtained from the Venom of the Scorpion *Centruroides bonito*

Rita Restano-Cassulini, Timoteo Olamendi-Portugal, Lidia Riaño-Umbarila, Fernando Z. Zamudio, Gustavo Delgado-Prudencio, Baltazar Becerril and Lourival D. Possani

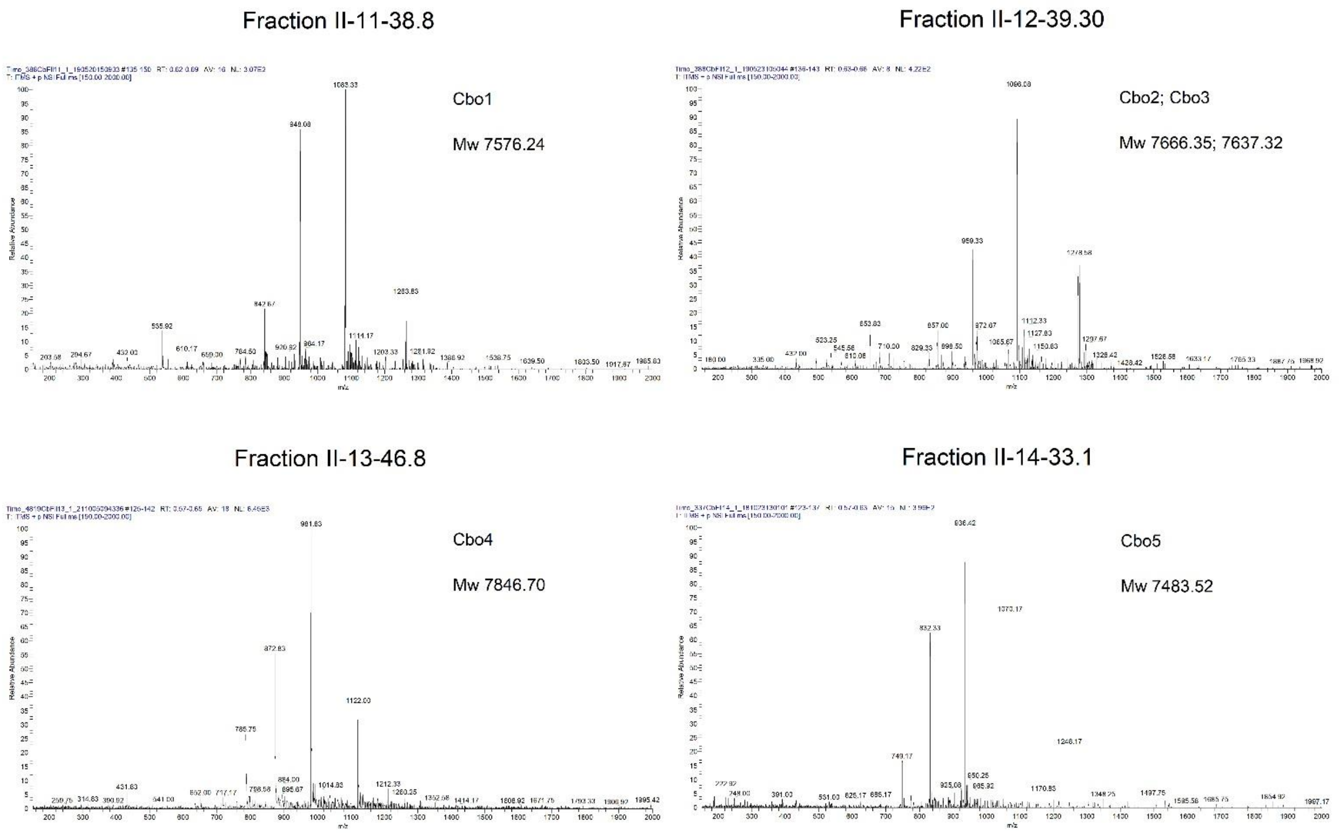

Figure S1. Mass spectrometry (MS) data. The mass spectrometry proteomics data have been deposited to the ProteomeXchange Consortium via the PRIDE partner repository with the dataset identifier PXD050138.

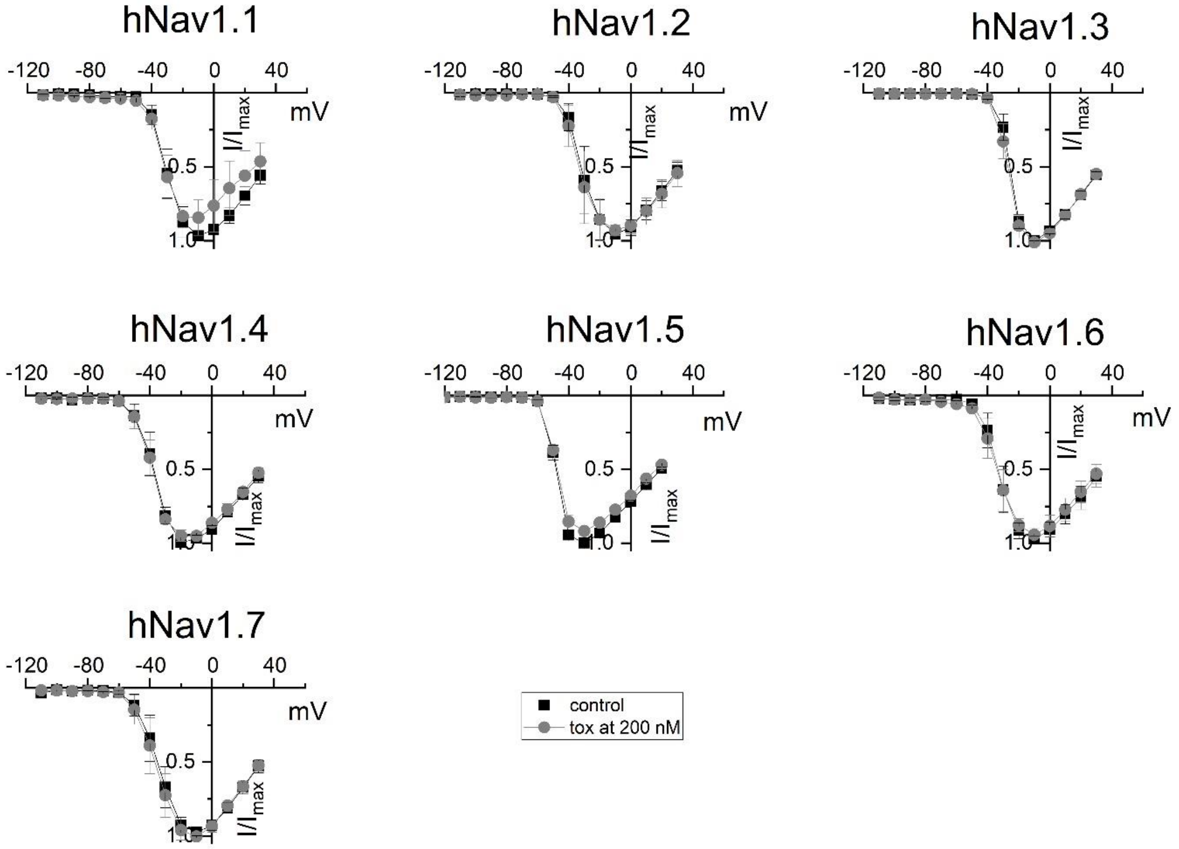

Figure S2. Voltage gated sodium channels hNav 1.1 – hNav 1.7 are insensible to Cbo5. Figure shows current / voltage relation graphs for sodium currents recorded in control and after exposition of 200 nM of Cbo5 for 30-60 s. Symbols are accordingly to legend. Data are the mean of 3-4 cells.

Table S1. Determination of LD_{50} of *C. bonito* venom using the "up and down" method.

**Dose (μg/mouse weighing 20 g)**	**Alive (L) or dead(D)**																**Total live**	$\boldsymbol{i}$
20	D												D				0	3
18		D				D		D				L		D		D	1	2
16			D		L		L		D		L				L		4	1
14				L						L							2	0
																	N =7	$\sum in_i$= 6

From the results reported in the table S1, the LD_{50} was determined using the formula described by Dixon and Mood:

$$LD_{50} = y' + d\left(\frac{A}{N} + \frac{1}{2}\right)$$

Where:

$$y' = 14\ \mu g$$
$$d = 2\ \ \mu g$$
$$N = 7$$
$$\sum in_i = 6$$

Resulting in:

$$LD_{50} = 14 + 2\left(\frac{6}{7} + \frac{1}{2}\right)$$

LD_{50} = 16.7 μg / mouse of the CD1 strain weighing 20 g

Table S2. Fitting parameters of data from activation, inactivation and total current of hNav 1.1-1.7 channels.

			hNav 1.1	hNav 1.2	hNav 1.3	hNav 1.4	hNav 1.5	hNav1.6	hNav 1.7
Cbo1	activation	$V_{0.5}$ (mV)	-24.3 ± 0.27	-21.86 ± 0.28	-20.26 ± 0.25	-24.03 ± 0.31	-42.28 ± 0.39	-22.52 ± 0.28	-18.37 ± 0.15
			-25.6 ± 0.23	-22.35 ± 0.3	-21.42 ± 0.26	-23.12 ± 0.28	-42.49 ± 0.36	-57.37 ± 1.69 (*)	-18.7 ± 0.19
		slope	8.65 ± 0.24	7.26 ± 0.25	7.43 ± 0.22	10.17 ± 0.27	5.91 ± 0.34	7.4 ± 0.25	8.35 ± 0.13
			8.95 ± 0.21	7.56 ± 0.26	7.7 ± 0.23	10.48 ± 0.25	5.77 ± 0.32	9.88 ± 1.07	8.5 ± 0.17
		A	n.c.	n.c.	n.c.	n.c.	n.c.	0.43 ± 0.03	n.c.
	inactivai ton	$V_{0.5}$ (mV)	-62.67 ± 0.28	-58.39 ± 0.19	-59.2 ± 0.51	-71.59 ± 0.33	-75.27 ± 0.27	-64 ± 0.57	-65.57 ± 0.27
			-65.21 ± 0.35	-58.88 ± 0.19	-59.49 ± 0.48	-71.73 ± 0.37	-74.59 ± 0.25	-68.51 ± 1.05	-65.02 ± 0.26
		slope	7.67 ± 0.25	6.88 ± 0.17	9.55 ± 0.45	8.42 ± 0.29	7.6 ± 0.24	7.22 ± 0.5	9.15 ± 0.24
			7.78 ± 0.31	6.78 ± 0.17	9.76 ± 0.42	8.25 ± 0.33	7.51 ± 0.22	10.04 ± 0.93	9.08 ± 0.23
		I/I_{max}	0.99 ± 0.02	0.98 ± 0.01	1.01 ± 0.01	0.89 ± 0.05	0.96 ± 0.01	0.75 ± 0.04 (*)	1.03 ± 0.03
		n	5	5	6	7	5	6	5
Cbo2+3	activation	$V_{0.5}$ (mV)	-29.11 ± 0.34	-30.27 ± 0.33	-29.26 ± 0.31	-27.57 ± 0.44	-36.17 ± 0.45	-20.39 ± 0.38	-10.41 ± 0.29
			-29.99 ± 0.37	-32.12 ± 0.27	-31.12 ± 0.38	-28.62 ± 0.46	-37.52 ± 0.33	-34.95 ± 14.6(*)	-10.43 ± 0.39
		slope	8.03 ± 0.3	7.99 ± 0.29	6.32 ± 0.28	10.03 ± 0.39	7.45 ± 0.4	-8 ± 0.34	7.98 ± 0.25
			7.99 ± 0.33	7.93 ± 0.24	6.02 ± 0.34	10.72 ± 0.4	7.31 ± 0.29	-10.97 ± 2.16	8.48 ± 0.34
		A	n.c.	n.c.	n.c	0.98 ± 0.01	n.c.	0.68 ± 0.3	n.c.
	inactivai ton	$V_{0.5}$ (mV)	-65.05 ± 0.39	-66.31 ± 0.16	-66.37 ± 0.21	-74.93 ± 0.38	-76.2 ± 0.31	-62.09 ± 0.6	-62.84 ± 0.21
			-65.93 ± 0.34	-66.72 ± 0.15	-67.55 ± 0.18	-76.21 ± 0.54	-77.21 ± 0.41	-63.91 ± 0.64	-63.15 ± 0.22
		slope	8.51 ± 0.34	7.4 ± 0.14	8.85 ± 0.19	7.54 ± 0.34	7.41 ± 0.27	6.91 ± 0.53	6.7 ±
			8.91 ± 0.3	7.51 ± 0.13	8.83 ± 0.16	8.53 ± 0.48	7.75 ± 0.36	7.1 ± 0.56	6.6 ±
		I/I_{max}	1.01 ± 0.01	1.03 ± 0.1	1.02 ± 0.01	0.84 ± 0.09	0.96 ± 0.01	0.99 ± 0.01	1.02 ± 0.01
		n	5	4	3	5	4	4	3
Cbo4	activation	$V_{0.5}$ (mV)	-21.12 ± 0.4	-20.37 ± 0.34	-27.55 ± 0.17	-29.4 ± 0.46	-45.17 ± 0.53	-29.55 ± 0.29	-16.41 ± 0.25
			-22.18 ± 0.38	-36.74 ± 4.08 (*)	-28.6 ± 0.22	-41.73 ± 1.44 (*)	-40.9 ± 0.5 (*)	-66.23 ± 3.14 (*)	-17.31 ± 0.25
		slope	7.86 ± 0.35	-7.47 ± 0.3	-9.01 ± 0.15	-7.04 ± 0.41	6.62 ± 0.46	-6.39 ± 0.25	7.14 ± 0.22
			8.57 ± 0.33	-16.75 ± 1.45 (*)	-9.08 ± 0.2	-15.87 ± 0.7 (*)	9.5 ± 0.44 (*)	-6.56 ± 2.49	7.44 ± 0.22
		A	n.c.	0.78 ± 0.04	n.c.	0.34 ± 0.06	n.c.	0.5 ± 0.06	n.c.
	inactivai ton	$V_{0.5}$ (mV)	-61.93 ± 0.23	-57.06 ± 0.4	-63.3 ± 0.38	-71.48 ± 0.14	-76.95 ± 0.3	-63.17 ± 0.37	-55.9 ± 0.41
			-63.79 ± 0.44	-59.21 ± 0.42	-63.57 ± 0.29	-74.82 ± 0.42	-72.7 ± 0.61	-65.67 ± 0.32	-55.8 ± 0.49
		slope	6.64 ± 0.2	6.5 ± 0.35	9.26 ± 0.34	6.95 ± 0.12	7.98 ± 0.27	6.53 ± 0.33	8.33 ± 0.36
			7 ± 0.39	7.49 ± 0.37	9.06 ± 0.25	8.91 ± 0.37	8.76 ± 0.54	6.94 ± 0.27	8.78 ± 0.43
		I/I_{max}	0.96 ± 0.02	0.9 ± 0.03	0.99 ± 0.01	0.48 ± 0.07 (*)	0.26 ± 0.04 (*)	0.44 ± 0.05 (*)	1.03 ± 0.01
		n	4	4	4	5	5	5	4

For activation and inactivation parameters, values of toxin treatment are in light grey shadow rows, while values of control are in no-shadow rows. $V_{0.5}$ (mV): membrane potential of middle activation or inactivation; A: fraction of channels unbound to the toxin; Ʈ (ms): time constant of the inactivation process, calculated at -10 mV; I/I_{max}: fraction of residual current after toxin application; n: number of recorded cells; n.c.: not calculated. Data indicates the mean of 3-7 cells ± standard error.

(*) mean significantly different at 0.05 level in a paired sample t Test.

Table S3. Plain text and FASTA format for toxins Cbo1 to Cbo5.

Protein ID	Name	Sequence	len
C0HMA3	Cbo1	KEGYLVNHSTGCKYECYKLGDNDYCLRECKQQYGKGAGGYCYAFGCWC-THLYEQAVVWPLPKKTCN	66
C0HMA4	Cbo2	KEGYIVNYHDGCKYECYKLGDNDYCLRECRARYGKGAGGYCYAFGCWC-THLYEQAVVWPLPKKTCN	66
C0HMA5	Cbo3	KEGYIVNYHDGCKYECYKLGDNDYCLRECKARYGKGAGGYCYAFGCWC-THLYEQAVVWPLPKKTCN	66
C0HMA6	Cbo4	KEGYIVDYHTGCKYTCAKLGDNDYCVRECRLRYYQSAHGYCYAFACWC-THLYEQAVVWPLPNKRCK	66
C0HMA7	Cbo5	KKDGYLVDKTGCKYTCWKLGENKYCNRECTWKHRGGNY-GYCYGFGCYCEGLADSTQTWPLPNKSC	65

>C0HMA3_Cbo1: KEGYLVNHSTGCKYECYKLGDNDYCLRECKQQYGKGAGGYCYAFGCWCTHLYEQAVVW-PLPKKTCN; >C0HMA4_Cbo2: KEGYIVNYHDGCKYECYKLGDNDYCLRECRARYGKGAGGYCYAFGCWCTHLYEQAVVW-PLPKKTCN; >C0HMA5_Cbo3: KEGYIVNYHDGCKYECYKLGDNDYCLRECKARYGKGAGGYCYAFGCWCTHLYEQAVVW-PLPKKTCN; >C0HMA6_Cbo4: KEGYIVDYHTGCKYTCAKLGDNDYCVRECRLRYYQSAHGYCYAFACWCTHLYEQAVVW-PLPNKRCK; >C0HMA7_Cbo5: KKDGYLVDKTGCKYTCWKLGENKYCNRECTWKHRGGNYGYCYGFGCYCEGLADSTQTW-PLPNKSC
